# 1-[2-(4-Isobutyl­phen­yl)propano­yl]thiosemicarbazide

**DOI:** 10.1107/S1600536809006527

**Published:** 2009-02-28

**Authors:** Hoong-Kun Fun, Reza Kia, Samuel Robinson Jebas, K. V. Sujith, B. Kalluraya

**Affiliations:** a X-ray Crystallography Unit, School of Physics, Universiti Sains Malaysia, 11800 USM, Penang, Malaysia; b Department of Studies in Chemistry, Mangalore University, Mangalagangotri, Mangalore 574 199, India

## Abstract

In the title compound, C_14_H_21_N_3_OS, inter­molecular N—H⋯O inter­actions generate ten-membered rings with *R*_2_^2^(10) ring motifs, whereas N—H⋯S inter­actions generate eight, 14- and 16-membered rings with *R*_2_^2^(8), *R*_4_^4^(14) and *R*_4_^4^(16) ring motifs, respectively. There are weak intra­molecular N—H⋯π inter­actions which might influence the conformation of the mol­ecule. The compound has a stereogenic center but the space group is centrosymmetic so the mol­ecule exists as a racemate.

## Related literature

For hydrogen-bond motifs, see: Bernstein *et al.* (1995 ▶). For biomedical applications of non-steroidal anti-inflammatory drugs, see, for example; Kawail *et al.* (2005 ▶); Klasser & Epstein (2005 ▶); Kean & Buchanan (2005 ▶); Nielsen & Bundgaard (1988 ▶); Khan & Akhter (2005 ▶); Zhao *et al.* (2006 ▶). For the stability of the temperature controller used in the data collection, see: Cosier & Glazer (1986 ▶).
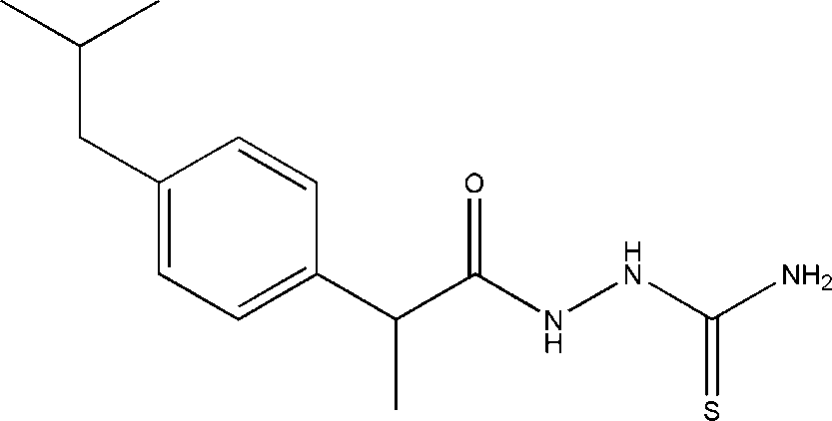


## Experimental

### 

#### Crystal data

C_14_H_21_N_3_OS
*M* *_r_* = 279.40
Triclinic, 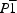

*a* = 5.5347 (1) Å
*b* = 10.6209 (3) Å
*c* = 13.1435 (3) Å
α = 97.935 (1)°
β = 98.418 (1)°
γ = 96.293 (1)°
*V* = 750.30 (3) Å^3^
*Z* = 2
Mo *K*α radiation
μ = 0.21 mm^−1^
*T* = 100 K
0.54 × 0.32 × 0.15 mm

#### Data collection

Bruker SMART APEXII CCD area-detector diffractometer
Absorption correction: multi-scan (**SADABS**; Bruker, 2005 ▶) *T*_min_ = 0.894, *T*_max_ = 0.969
18596 measured reflections
6524 independent reflections
5896 reflections with *I* > 2σ(*I*)
*R*_int_ = 0.018

#### Refinement

*R*[*F* ^2^ > 2σ(*F* ^2^)] = 0.034
*wR*(*F* ^2^) = 0.096
*S* = 1.05
6524 reflections
191 parameters
H atoms treated by a mixture of independent and constrained refinement
Δρ_max_ = 0.68 e Å^−3^
Δρ_min_ = −0.32 e Å^−3^

### 

Data collection: *APEX2* (Bruker, 2005 ▶); cell refinement: *SAINT* (Bruker, 2005 ▶); data reduction: *SAINT*; program(s) used to solve structure: *SHELXTL* (Sheldrick, 2008 ▶); program(s) used to refine structure: *SHELXTL*; molecular graphics: *SHELXTL*; software used to prepare material for publication: *SHELXTL* and *PLATON* (Spek, 2009 ▶).

## Supplementary Material

Crystal structure: contains datablocks global, I. DOI: 10.1107/S1600536809006527/dn2428sup1.cif

Structure factors: contains datablocks I. DOI: 10.1107/S1600536809006527/dn2428Isup2.hkl

Additional supplementary materials:  crystallographic information; 3D view; checkCIF report

## Figures and Tables

**Table 1:** Hydrogen-bond geometry (Å, °)

*D*—H⋯*A*	*D*—H	H⋯*A*	*D*⋯*A*	*D*—H⋯*A*
N2—H1*N*2⋯O1^i^	0.857 (15)	2.029 (15)	2.8745 (9)	169.0 (14)
N1—H1*N*1⋯S1^ii^	0.886 (12)	2.495 (13)	3.3324 (7)	157.8 (11)
N3—H1*N*3⋯S1^iii^	0.842 (15)	2.577 (15)	3.3945 (7)	164.1 (14)
C7—H7*A*⋯O1^ii^	1.00	2.44	3.3501 (9)	151
N3—H2*N*3⋯*Cg*1	0.850 (16)	2.870 (14)	3.5083 (7)	133.4 (12)

Symmetry codes: (i) 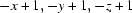; (ii) 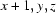; (iii) 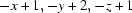. *Cg*1 is the centroid of the C1–C6 benzene ring.

**Fig. 1.**
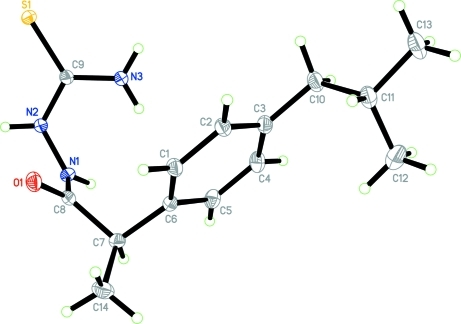
Molecular structure of (I) with the atom labeling scheme. Ellipsoids are drawn at the 50% probability level. H atoms are represented as small sphere of arbitrary radii. The enantiomer represented has R configuration at C7.

**Fig. 2.**
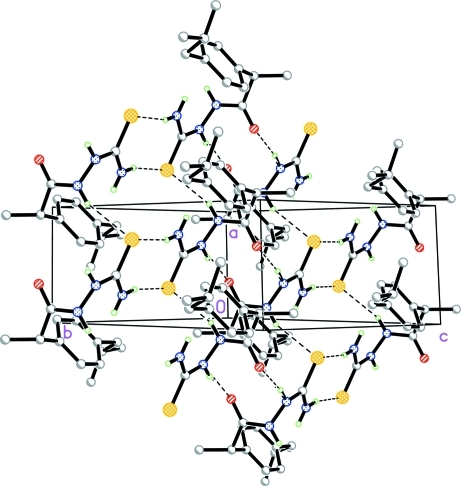
The crystal packing of (I) showing the graph set motifs. Intermolecular interactions are shown as dashed lines. H atoms not involved in hydrogen bondings have been removed for clarity.

## supplementary crystallographic information

### Comment

Non-steroidal anti-inflammatory drugs (NSAIDs) such as ibuprofen are widely used in the treatment of pain and inflammation (Kawail *et al.*, 2005; Klasser & Epstein, 2005).In fact, prolonged use of NSAIDs, for example ibuprofen, has been associated with gastrointestinal complications (Kean & Buchanan, 2005). Therefore, synthetic approaches based upon NSAIDs chemical modification have been undertaken with the aim of improving the NSAID safety profile. The utilization of prodrugs to temporarily mask the acidic group of NSAIDs has been proposed as an approach to reduce or suppress the GI toxicity due to the direct contact effect and also to increase their absorption values (Nielsen & Bundgaard, 1988). Ester prodrugs of ibuprofen have been synthesized with this aim (Khan & Akhter, 2005). Ester prodrugs of ibuprofen were synthesized and found to have anti-inflammatory, analgesic and ulcerogenic activities (Zhao *et al.*, 2006). Due to these reasons, we have synthesized the thiosemicarbazide analogue of ibuprofen and report its crystal structure.

The title compound, I, Fig. 1, comprises a single molecule in the asymmetric unit. Intermolecular N—H···O interactions generate ten-membered rings producing *R*_2_^2^(10) ring motifs, whereas N—H···S interactions generate eight, fourteen, sixteen rings producing *R*_2_^2^(8), *R*_4_^4^(14) and *R*_4_^4^(16) ring motifs, respectively. (Fig.2) (Bernstein *et al.*, 1995). There is a weak intramolecular N—H···π interaction (Table 1, *Cg*1 is the centroid of the C1–C6 benzene ring). The compound has a stereogenic center at C7 but the space group is centrosymmetic so the molecule exists as a racemate.

### Experimental

A mixture of 2-[2-(4-isobutylphenyl)propanoyl]hydrazine (0.01 mole), potassium thiocyanate (1.9 g, 0.02 mole), conc. HCl (1 ml) and water (20 ml) was refluxed for 3 h. On cooling the solid obtained was collected by filtration, washed with water and dried. Crystals suitable for *X*-ray analysis were obtained from ethanol by slow evaporation (Yield 62%; m.p. 447 K).

### Refinement

N-bound hydrogen atoms were located from the difference Fourier map and refined freely; see Table 1. The rest of the hydrogen atoms were positioned geometrically and constrained to refine with the parent atoms with C—H = 0.93–1.00 Å and *U*_iso_(H) = 1.2 or 1.5 *U*_eq_(C). A rotating group model was used for the methyl groups.

### Crystal data

**Table:**

C_14_H_21_N_3_OS	*Z* = 2
*M**_r_* = 279.40	*F*(000) = 300
Triclinic, *P*1	*D*_x_ = 1.237 Mg m^−^^3^
Hall symbol: -P 1	Melting point: 335 K
*a* = 5.5347 (1) Å	Mo *K*α radiation, λ = 0.71073 Å
*b* = 10.6209 (3) Å	Cell parameters from 9906 reflections
*c* = 13.1435 (3) Å	θ = 2.3–38.2°
α = 97.935 (1)°	µ = 0.21 mm^−^^1^
β = 98.418 (1)°	*T* = 100 K
γ = 96.293 (1)°	Block, colourless
*V* = 750.30 (3) Å^3^	0.54 × 0.32 × 0.15 mm

### Data collection

**Table:**

Bruker SMART APEXII CCD area-detector diffractometer	6524 independent reflections
Radiation source: fine-focus sealed tube	5896 reflections with *I* > 2σ(*I*)
graphite	*R*_int_ = 0.018
φ and ω scans	θ_max_ = 35.0°, θ_min_ = 1.6°
Absorption correction: multi-scan (*SADABS*; Bruker, 2005)	*h* = −8→8
*T*_min_ = 0.894, *T*_max_ = 0.969	*k* = −16→17
18596 measured reflections	*l* = −20→21

### Refinement

**Table:**

Refinement on *F*^2^	Primary atom site location: structure-invariant direct methods
Least-squares matrix: full	Secondary atom site location: difference Fourier map
*R*[*F*^2^ > 2σ(*F*^2^)] = 0.034	Hydrogen site location: inferred from neighbouring sites
*wR*(*F*^2^) = 0.096	H atoms treated by a mixture of independent and constrained refinement
*S* = 1.05	*w* = 1/[σ^2^(*F*_o_^2^) + (0.0494*P*)^2^ + 0.1966*P*] where *P* = (*F*_o_^2^ + 2*F*_c_^2^)/3
6524 reflections	(Δ/σ)_max_ = 0.001
191 parameters	Δρ_max_ = 0.68 e Å^−^^3^
0 restraints	Δρ_min_ = −0.32 e Å^−^^3^

### Special details

**Table:**

Experimental. The crystal was placed in the cold stream of an Oxford Cryosystems Cobra open-flow nitrogen cryostat (Cosier & Glazer, 1986) operating at 100.0 (1) K.
Geometry. All e.s.d.'s (except the e.s.d. in the dihedral angle between two l.s. planes) are estimated using the full covariance matrix. The cell e.s.d.'s are taken into account individually in the estimation of e.s.d.'s in distances, angles and torsion angles; correlations between e.s.d.'s in cell parameters are only used when they are defined by crystal symmetry. An approximate (isotropic) treatment of cell e.s.d.'s is used for estimating e.s.d.'s involving l.s. planes.
Refinement. Refinement of *F*^2^ against ALL reflections. The weighted *R*-factor *wR* and goodness of fit *S* are based on *F*^2^, conventional *R*-factors *R* are based on *F*, with *F* set to zero for negative *F*^2^. The threshold expression of *F*^2^ > σ(*F*^2^) is used only for calculating *R*-factors(gt) *etc*. and is not relevant to the choice of reflections for refinement. *R*-factors based on *F*^2^ are statistically about twice as large as those based on *F*, and *R*- factors based on ALL data will be even larger.

### Fractional atomic coordinates and isotropic or equivalent isotropic displacement parameters (Å^2^)

**Table:**

	*x*	*y*	*z*	*U*_iso_*/*U*_eq_	
S1	0.30182 (3)	0.806105 (17)	0.407445 (14)	0.01523 (5)	
O1	0.65430 (10)	0.57112 (5)	0.62717 (5)	0.01802 (10)	
N1	0.87208 (11)	0.64109 (6)	0.50767 (5)	0.01429 (10)	
N2	0.66238 (12)	0.66716 (6)	0.44588 (5)	0.01530 (11)	
N3	0.74599 (12)	0.87876 (6)	0.52340 (5)	0.01701 (11)	
C1	0.95446 (14)	0.81565 (7)	0.78524 (6)	0.01651 (12)	
H1A	0.8281	0.7578	0.8019	0.020*	
C2	0.96885 (14)	0.94657 (7)	0.82013 (6)	0.01724 (12)	
H2A	0.8519	0.9769	0.8604	0.021*	
C3	1.15276 (14)	1.03429 (7)	0.79679 (6)	0.01707 (12)	
C4	1.32484 (15)	0.98630 (8)	0.73933 (6)	0.01981 (14)	
H4A	1.4534	1.0439	0.7239	0.024*	
C5	1.31130 (14)	0.85529 (8)	0.70412 (6)	0.01828 (13)	
H5A	1.4303	0.8249	0.6650	0.022*	
C6	1.12491 (13)	0.76844 (7)	0.72571 (5)	0.01440 (11)	
C7	1.09656 (13)	0.62723 (7)	0.67782 (6)	0.01577 (12)	
H7A	1.2328	0.6134	0.6369	0.019*	
C8	0.85365 (13)	0.60673 (6)	0.60322 (6)	0.01385 (11)	
C9	0.58753 (12)	0.78460 (6)	0.46377 (5)	0.01334 (11)	
C10	1.16404 (17)	1.17610 (7)	0.83421 (6)	0.02134 (14)	
H10A	0.9976	1.2013	0.8173	0.026*	
H10B	1.2750	1.2240	0.7960	0.026*	
C11	1.25548 (16)	1.21505 (8)	0.95183 (6)	0.02091 (14)	
H11A	1.1465	1.1631	0.9893	0.025*	
C12	1.5176 (2)	1.18766 (13)	0.98222 (10)	0.0422 (3)	
H12A	1.5659	1.2084	1.0580	0.063*	
H12B	1.6290	1.2402	0.9485	0.063*	
H12C	1.5271	1.0967	0.9598	0.063*	
C13	1.2345 (2)	1.35641 (8)	0.98524 (8)	0.02918 (18)	
H13A	1.2943	1.3802	1.0601	0.044*	
H13B	1.0617	1.3706	0.9700	0.044*	
H13C	1.3339	1.4091	0.9470	0.044*	
C14	1.09915 (18)	0.53538 (8)	0.75758 (7)	0.02360 (15)	
H14A	1.2581	0.5521	0.8044	0.035*	
H14B	1.0747	0.4467	0.7214	0.035*	
H14C	0.9664	0.5484	0.7982	0.035*	
H1N2	0.556 (3)	0.6019 (14)	0.4190 (11)	0.027 (3)*	
H1N1	1.013 (2)	0.6698 (13)	0.4891 (10)	0.024 (3)*	
H2N3	0.888 (3)	0.8658 (13)	0.5509 (11)	0.029 (3)*	
H1N3	0.703 (3)	0.9524 (14)	0.5348 (11)	0.027 (3)*	

### Atomic displacement parameters (Å^2^)

**Table:**

	*U*^11^	*U*^22^	*U*^33^	*U*^12^	*U*^13^	*U*^23^
S1	0.01232 (8)	0.01386 (8)	0.01930 (9)	0.00173 (5)	0.00039 (6)	0.00418 (6)
O1	0.0155 (2)	0.0151 (2)	0.0225 (3)	−0.00111 (18)	0.00474 (19)	0.00054 (19)
N1	0.0111 (2)	0.0143 (2)	0.0172 (2)	0.00244 (18)	0.00100 (19)	0.00253 (19)
N2	0.0134 (2)	0.0117 (2)	0.0190 (3)	0.00211 (19)	−0.0017 (2)	0.00032 (19)
N3	0.0137 (2)	0.0116 (2)	0.0239 (3)	0.00168 (19)	−0.0009 (2)	0.0003 (2)
C1	0.0146 (3)	0.0140 (3)	0.0202 (3)	0.0002 (2)	0.0040 (2)	0.0003 (2)
C2	0.0164 (3)	0.0145 (3)	0.0197 (3)	0.0017 (2)	0.0028 (2)	−0.0006 (2)
C3	0.0196 (3)	0.0145 (3)	0.0151 (3)	−0.0006 (2)	−0.0012 (2)	0.0020 (2)
C4	0.0206 (3)	0.0196 (3)	0.0175 (3)	−0.0040 (3)	0.0034 (2)	0.0018 (2)
C5	0.0153 (3)	0.0213 (3)	0.0169 (3)	−0.0004 (2)	0.0033 (2)	−0.0002 (2)
C6	0.0127 (3)	0.0145 (3)	0.0149 (3)	0.0020 (2)	0.0002 (2)	0.0005 (2)
C7	0.0147 (3)	0.0148 (3)	0.0173 (3)	0.0043 (2)	0.0008 (2)	0.0007 (2)
C8	0.0141 (3)	0.0098 (2)	0.0168 (3)	0.0020 (2)	0.0019 (2)	−0.0004 (2)
C9	0.0128 (3)	0.0121 (2)	0.0152 (3)	0.0012 (2)	0.0025 (2)	0.0026 (2)
C10	0.0288 (4)	0.0131 (3)	0.0194 (3)	−0.0001 (3)	−0.0021 (3)	0.0019 (2)
C11	0.0263 (4)	0.0156 (3)	0.0185 (3)	−0.0009 (3)	0.0011 (3)	0.0004 (2)
C12	0.0356 (5)	0.0422 (6)	0.0389 (6)	0.0109 (5)	−0.0156 (4)	−0.0103 (5)
C13	0.0385 (5)	0.0165 (3)	0.0286 (4)	−0.0021 (3)	0.0034 (4)	−0.0032 (3)
C14	0.0315 (4)	0.0181 (3)	0.0212 (3)	0.0078 (3)	0.0000 (3)	0.0044 (3)

### Geometric parameters (Å, °)

**Table:**

S1—C9	1.6982 (7)	C5—H5A	0.9500
O1—C8	1.2259 (9)	C6—C7	1.5261 (10)
N1—C8	1.3699 (10)	C7—C8	1.5190 (10)
N1—N2	1.3927 (9)	C7—C14	1.5270 (11)
N1—H1N1	0.884 (14)	C7—H7A	1.0000
N2—C9	1.3578 (9)	C10—C11	1.5381 (12)
N2—H1N2	0.855 (15)	C10—H10A	0.9900
N3—C9	1.3318 (9)	C10—H10B	0.9900
N3—H2N3	0.851 (14)	C11—C12	1.5178 (14)
N3—H1N3	0.843 (14)	C11—C13	1.5269 (12)
C1—C2	1.3925 (10)	C11—H11A	1.0000
C1—C6	1.4019 (10)	C12—H12A	0.9800
C1—H1A	0.9500	C12—H12B	0.9800
C2—C3	1.4002 (11)	C12—H12C	0.9800
C2—H2A	0.9500	C13—H13A	0.9800
C3—C4	1.3957 (12)	C13—H13B	0.9800
C3—C10	1.5100 (11)	C13—H13C	0.9800
C4—C5	1.3947 (11)	C14—H14A	0.9800
C4—H4A	0.9500	C14—H14B	0.9800
C5—C6	1.3946 (11)	C14—H14C	0.9800
			
C8—N1—N2	119.30 (6)	N1—C8—C7	114.12 (6)
C8—N1—H1N1	123.8 (9)	N3—C9—N2	117.81 (6)
N2—N1—H1N1	114.5 (8)	N3—C9—S1	123.05 (5)
C9—N2—N1	119.39 (6)	N2—C9—S1	119.12 (5)
C9—N2—H1N2	119.6 (9)	C3—C10—C11	113.56 (6)
N1—N2—H1N2	115.4 (9)	C3—C10—H10A	108.9
C9—N3—H2N3	121.5 (10)	C11—C10—H10A	108.9
C9—N3—H1N3	118.9 (10)	C3—C10—H10B	108.9
H2N3—N3—H1N3	119.6 (13)	C11—C10—H10B	108.9
C2—C1—C6	120.56 (7)	H10A—C10—H10B	107.7
C2—C1—H1A	119.7	C12—C11—C13	110.83 (8)
C6—C1—H1A	119.7	C12—C11—C10	111.70 (8)
C1—C2—C3	121.12 (7)	C13—C11—C10	110.21 (7)
C1—C2—H2A	119.4	C12—C11—H11A	108.0
C3—C2—H2A	119.4	C13—C11—H11A	108.0
C4—C3—C2	117.97 (7)	C10—C11—H11A	108.0
C4—C3—C10	121.57 (7)	C11—C12—H12A	109.5
C2—C3—C10	120.45 (7)	C11—C12—H12B	109.5
C5—C4—C3	121.16 (7)	H12A—C12—H12B	109.5
C5—C4—H4A	119.4	C11—C12—H12C	109.5
C3—C4—H4A	119.4	H12A—C12—H12C	109.5
C6—C5—C4	120.71 (7)	H12B—C12—H12C	109.5
C6—C5—H5A	119.6	C11—C13—H13A	109.5
C4—C5—H5A	119.6	C11—C13—H13B	109.5
C5—C6—C1	118.45 (7)	H13A—C13—H13B	109.5
C5—C6—C7	120.22 (6)	C11—C13—H13C	109.5
C1—C6—C7	121.14 (6)	H13A—C13—H13C	109.5
C8—C7—C6	104.02 (5)	H13B—C13—H13C	109.5
C8—C7—C14	112.04 (7)	C7—C14—H14A	109.5
C6—C7—C14	113.91 (6)	C7—C14—H14B	109.5
C8—C7—H7A	108.9	H14A—C14—H14B	109.5
C6—C7—H7A	108.9	C7—C14—H14C	109.5
C14—C7—H7A	108.9	H14A—C14—H14C	109.5
O1—C8—N1	121.87 (7)	H14B—C14—H14C	109.5
O1—C8—C7	123.83 (7)		
			
C8—N1—N2—C9	81.80 (8)	C1—C6—C7—C14	−59.88 (9)
C6—C1—C2—C3	0.06 (12)	N2—N1—C8—O1	16.17 (10)
C1—C2—C3—C4	−1.36 (11)	N2—N1—C8—C7	−159.10 (6)
C1—C2—C3—C10	179.31 (7)	C6—C7—C8—O1	−91.51 (8)
C2—C3—C4—C5	1.40 (12)	C14—C7—C8—O1	31.97 (10)
C10—C3—C4—C5	−179.28 (7)	C6—C7—C8—N1	83.65 (7)
C3—C4—C5—C6	−0.13 (12)	C14—C7—C8—N1	−152.87 (6)
C4—C5—C6—C1	−1.19 (11)	N1—N2—C9—N3	14.79 (10)
C4—C5—C6—C7	173.91 (7)	N1—N2—C9—S1	−166.70 (5)
C2—C1—C6—C5	1.22 (11)	C4—C3—C10—C11	−106.27 (9)
C2—C1—C6—C7	−173.83 (7)	C2—C3—C10—C11	73.03 (10)
C5—C6—C7—C8	−112.59 (7)	C3—C10—C11—C12	62.58 (11)
C1—C6—C7—C8	62.37 (8)	C3—C10—C11—C13	−173.75 (8)
C5—C6—C7—C14	125.16 (8)		

### Hydrogen-bond geometry (Å, °)

**Table:**

*D*—H···*A*	*D*—H	H···*A*	*D*···*A*	*D*—H···*A*
N2—H1N2···O1^i^	0.857 (15)	2.029 (15)	2.8745 (9)	169.0 (14)
N1—H1N1···S1^ii^	0.886 (12)	2.495 (13)	3.3324 (7)	157.8 (11)
N3—H1N3···S1^iii^	0.842 (15)	2.577 (15)	3.3945 (7)	164.1 (14)
C7—H7A···O1^ii^	1.00	2.44	3.3501 (9)	151
N3—H2N3···Cg1	0.850 (16)	2.870 (14)	3.5083 (7)	133.4 (12)

Symmetry codes: (i) −*x*+1, −*y*+1, −*z*+1; (ii) *x*+1, *y*, *z*; (iii) −*x*+1, −*y*+2, −*z*+1.

## References

[bb1] Bernstein, J., Davis, R. E., Shimoni, L. & Chang, N.-L. (1995). *Angew. Chem. Int. Ed. Engl.* **34**, 1555–1573.

[bb2] Bruker (2005). *APEX2*, *SAINT* and *SADABS* Bruker AXS Inc., Madison, Wisconsin, USA.

[bb14] Cosier, J. & Glazer, A. M. (1986). *J. Appl. Cryst.* **19**, 105–107.

[bb3] Kawail, S., Kojima, F. & Kusunoki, N. (2005). *Allergol. Int.* **54**, 209–215.

[bb4] Kean, W. F. & Buchanan, W. W. (2005). *Inflammopharmacology*, **13**, 343–370. 10.1163/156856005774415565 16354389

[bb5] Khan, M. S. Y. & Akhter, M. (2005). *Eur. J. Med. Chem.* **40**, 371–376. 10.1016/j.ejmech.2004.11.009 15804536

[bb6] Klasser, G. D. & Epstein, J. (2005). *J. Can. Dent. Assoc.* **71**, 575–580. 16202197

[bb7] Nielsen, N. M. & Bundgaard, H. (1988). *J. Pharm. Sci.* **77**, 285–298. 10.1002/jps.2600770402 3379586

[bb8] Sheldrick, G. M. (2008). *Acta Cryst.* A**64**, 112–122. 10.1107/S0108767307043930 18156677

[bb9] Spek, A. L. (2009). *Acta Cryst.* D**65**, 148–155. 10.1107/S090744490804362X PMC2631630 19171970

[bb10] Zhao, X., Tao, X., Wei, D. & Song, Q. (2006). *Eur. J. Med. Chem.* **41**, 1352–1358. 10.1016/j.ejmech.2006.05.014 16806590

